# F-box proteins in epigenetic regulation of cancer

**DOI:** 10.18632/oncotarget.22469

**Published:** 2017-11-16

**Authors:** Jia Shen, Charles Spruck

**Affiliations:** ^1^ Tumor Initiation and Maintenance Program, NCI-Designated Cancer Center, Sanford Burnham Prebys Medical Discovery Institute, San Diego, California, USA

**Keywords:** F-box protein, SCF, E3 ubiquitin ligase, epigenetics, cancer

## Abstract

Epigenetic abnormalities are now realized as important as genetic alterations in contributing to the initiation and progression of cancer. Recent advancements in the cancer epigenetics field have identified extensive alterations of the epigenetic network in human cancers, including histone modifications and DNA methylation. F-box proteins, the substrate receptors of SCF (SKP1-Cullin1-F-box protein) E3 ubiquitin ligases, can directly and indirectly affect the balance of epigenetic regulation. In this brief review, we discuss our current understanding of F-box proteins in cellular epigenetic regulation and how dysregulation of these processes contribute to cancer development.

## INTRODUCTION

Cancer poses a rising threat to human health, with the estimated number of Americans with a history of cancer predicted to surpass 20 million within the next 10 years [1–3]. Cancer cells harbor global epigenetic alterations, which cooperate with genetic mutations in tumor development [4, 5]. Epigenetic abnormalities include global changes in DNA methylation, histone modification patterns, and altered expression profiles of chromatin-modifying enzymes. These changes contribute to the inappropriate activation or inhibition of various signaling pathways, which participate in cancer initiation, progression, invasion, and metastasis [6–8].

It is well documented that F-box proteins function as substrate receptors for the SCF-type E3 ubiquitin ligase, which plays important roles in regulation of cell proliferation [9, 10], migration and invasion [11–13], metabolism [14, 15], angiogenesis [16, 17], cell death [18–20], and DNA damage response [21–23]. In addition, recent studies have reported putative roles for several F-box proteins in cellular epigenetic regulation, and dysregulation of these F-box proteins and their associated functions could contribute to tumorigenesis. In this review, we provide an overview, based on our current knowledge, of F-box proteins in the regulation of cancer epigenetics.

### Classification: the F-box protein families

**Figure 1:**
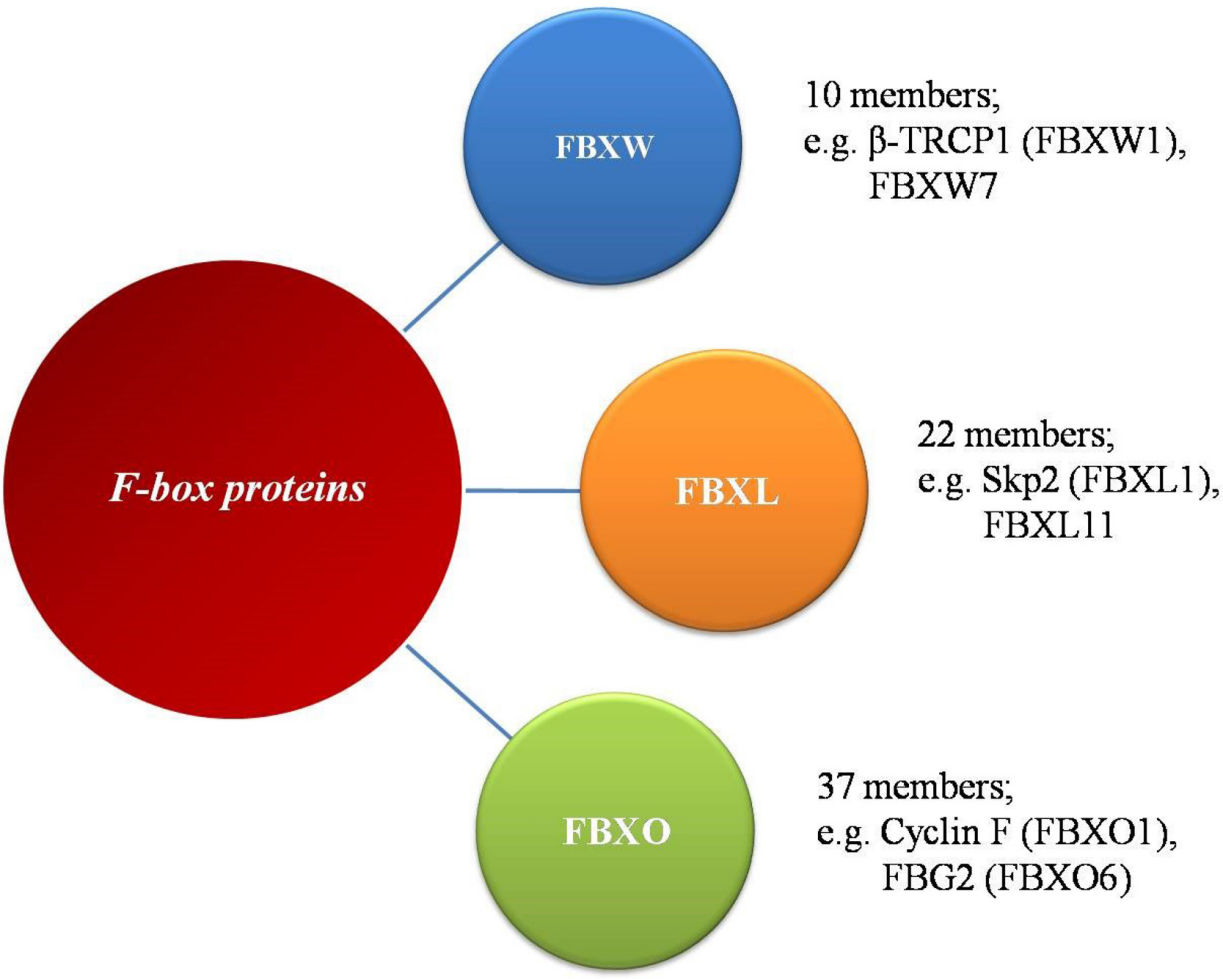
The family of F-box proteins The human F-box proteins have been classified into three subgroups according to specific substrate recognition domains.

In mammals, the largest family of E3 ubiquitin ligases is the Cullin-RING ligases (CRLs), of which SCF ligases are the best characterized. Humans express 69 different F-box proteins, which are categorized into three sub-families based on the presence of defined domains (Figure 1): FBXW- WD40 repeats, FBXL- leucine-rich repeats, and FBXO- undefined domains [24]. The FBXW subfamily is composed of 10 proteins, all of which contain WD40 repeat domains, including the well-studied β-TRCP1 (also known as FBXW1), FBXW7 (hCDC4) and β-TRCP2 (FBXW11). This subclass of F-box proteins mainly targets substrates involved in cell cycle regulation and tumorigenesis. The FBXL proteins, including SKP2 (also known as FBXL1), contain an F-box motif and a C-terminal Leu-rich repeat domain. This subgroup has 22 members that could be tumor suppressors or oncoproteins. The remaining 37 F-box proteins are designated as FBXO proteins and make up the largest subfamily of F-box proteins. FBXO proteins contain the F-box motif and different uncharacterized functional domains. The protein-protein interaction domains of F-box proteins mediate substrate recognition, with each F-box protein targeting a unique set of substrates, each harboring unique ‘degron’ motifs.

### Functions: F-box proteins in regulation of cancer epigenetics

Epigenetic alterations promote altered gene function, contributing to malignant cellular transformation. F-box proteins have been shown to directly and indirectly affect cellular epigenetics. FBXL10 (also known as Ndy1 or KDM2B), an F-box protein that binds CpG-rich promoters in the mammalian genome, has been shown to exert both histone ubiquitylation and histone demethylase activities [25, 26]. FBXL10 contains an N-terminal Jumonji C (JmjC) domain, followed by a CXXC zinc finger, a plant homeodomain finger (PHD), an F-box domain, and 8 leucine-rich repeats. Several lines of evidence have shown FBXL10 is overexpressed in many human cancers including acute myeloid leukemia (AML) [27], seminomas [28] and pancreatic ductal adenocarcinomas [29]. Kottakis *et al*. [30] showed knockdown of FBXL10 expression in a panel of human tumor-derived cell lines induced G_1_ phase delay and senescence and/or apoptosis. In addition, forced overexpression of FBXL10 in hematopoietic stem cells caused an acceleration of the G_0_/G_1_ to S phase transition and development of myeloid or B-lymphoid leukemia [31]. FBXL10 was shown to directly bind to CpG islands throughout the genome via its CXXC motif, and interact with Ring1B and Nspc1 to form a non-canonical Polycomb Repressive Complex 1 (PRC1), which lead to the gene repressive modification H2AK119 mono-ubiquitination (H2AK119ub1) [25, 32]. H2AK119ub1 is significantly correlated with poorer prognosis in patients with pancreatic ductal adenocarcinoma [25, 32]. PRC1-dependent H2AK119ub1 also leads to recruitment of PRC2 and trimethylation of histone H3 on lysine 27 (H3K27me3) [33], which is a pivotal mark in the establishment of repressive chromatin. FBXL10 expression promotes cell proliferation and bypass of the senescence barrier in primary cells, in part by counteracting the senescence-associated down-regulation of EZH2, a PRC2 component, leading to global and Ink4a/Arf locus-specific up-regulation of histone H3K27me3 [34–36]. On the other hand, depletion of FBXL10 was shown to cause upregulation of Arf in MEFs, which suggests FBXL10 might accelerate cell proliferation by inhibiting the Arf tumor-suppressor pathway. In addition, FBXL10 was found to demethylate histone H3 dimethylated at lysine 36 (H3K36me2), which is required for initiation and maintenance of acute myeloid leukemia. He *et al*. [37] found FBXL10 decreases transcription of the tumor suppressor p15Ink4b through demethylation of H3K36me2 near the gene promoter. Tzatsos *et al*. [29] demonstrated another potential mechanism by which FBXL10 could drive tumorigenesis. Utilizing gene expression arrays and ChIP assays, they showed FBXL10 repressed developmental genes by interacting with Polycomb Group (PcG) proteins at transcriptional start sites, and activated mediators of protein synthesis and mitochondrial function genes by interacting with the MYC oncogene and another histone demethylase FBXL11 (also known as KDM2B and JHDM1A).

FBXL11 and FBXL10 share conserved JmjC and CXXC domains, both of which can catalyze the demethylation of H3K36me2. Like FBXL10, FBXL11 can also bind CpG islands, though it preferentially recognizes non-methylated CpG DNA, and binding is interrupted by CpG methylation [38]. In addition, FBXL11 does not associate with PcG proteins so its function appears different from that of FBXL10. FBXL11 is frequently overexpressed in non-small cell lung cancers (NSCLCs) and this is correlated with poor prognosis [39]. FBXL11 was also shown to be indispensable for tumorigenicity and invasiveness of FBXL11-overexpressing NSCLC cells, and knockdown of FBXL11 expression decreased the growth and invasive capabilities of NSCLC cells in mouse xenograft models. Mechanistically, FBXL11 was shown to activate ERK1/2 through epigenetic repression of dual-specificity phosphatase 3 (DUSP3) via demethylation of H3K36me2 [39]. Another study showed FBXL11 transcriptionally repressed histone deacetylase 3 (HDAC3) through demethylation of H3K36me2 in FBXL11-overexpressing NSCLC cells [40]. Additionally, analysis of FBXL11 knockout mice showed its depletion resulted in significant loss of H2A ubiquitylation, indicating an important role in regulation of histone ubiquitination [41]. However, it is unclear how the ubiquitylation functions of FBXL10 and FBXL11 coordinate with their demethylase activities, though they are the only F-box proteins known to exhibit demethylase activity [42].

In addition to directly binding chromatin to regulate epigenetic modifications, F-box proteins can also indirectly influence cancer epigenetics through the direct targeting of epigenetic regulators for ubiquitin-dependent proteolysis. KDM4A (also known as JMJD2A) is a demethylase that targets histone H3K9me2/3 and H3K36me2/3 leading to transcriptional activation. KDM4A has been shown to play an important role in gene expression [43], cellular differentiation [44, 45] and cancer [46]. Tan *et al*. [47] and Van Rechem *et al*. [48] showed that FBXO22 and FBXL4 independently regulate KDM4A proteolysis. FBXO22 regulates cellular histone H3 marks and KDM4A target gene transcription by controlling KDM4A protein levels. FBXO22 depletion was shown to stabilize KDM4A resulting in a significant reduction in the abundance of H3K9me3 and H3K36me3 on promoters of KDM4A's target genes. Since KDM4A plays a role in cancer development, FBXO22 seems to be a tumor suppressor. Furthermore, the F-box protein β-TrCP1 (FBXW1A) mediates the ubiquitin-dependent proteolysis of UHRF1, which plays a critical role in maintaining DNA methylation patterns during DNA replication and its deregulated expression correlates with cancer development. In this case, β-TrCP1 exerts its tumor suppressor function to maintain genomic stability by targeting UHRF1 degradation in response to UV-induced DNA damage [49].

## DISCUSSION AND CONCLUSIONS

Our current knowledge demonstrates F-box proteins play pivotal roles in the epigenetic regulation of cancer, mediated through E3 ubiquitylation-dependent or -independent mechanisms (Table 1). Since only a handful of epigenetic regulator F-box proteins have been functionally characterized, research in this field is limited and many key questions remain unaddressed. For example: Do additional F-box proteins also regulate cancer epigenetic regulation? What are the upstream signaling pathways that control the functions of F-box proteins involved in regulation of cancer epigenetics? How do the epigenetic regulatory functions of F-box proteins coordinate with their ubiquitylation functions? More importantly, the growing understanding of how F-box proteins target epigenetic regulators will facilitate the development of cancer therapeutics that target this protein family. Although challenging, pharmacological inhibitors of few F-box proteins have been developed, and show significant promise. For example, inactivation of the SCF^SKP2^ ligase by small molecule inhibitors has been shown to have therapeutic potential. This should serve to instigate researchers to search for small molecules that target oncogenic F-box proteins involved in cancer epigenetics.

**Table 1:** F-box proteins in regulation of cancer epigenetics

F-box proteins	Targets	Functions	References
FBXL10	CpG islands and histone	Histone H2A ubiquitylation and histone H3 demethylation	[25, 26, 32, 37]
FBXL11	CpG islands and histone	Histone H2A ubiquitylation and histone H3 demethylation	[39, 40, 41]
FBXO22	KDM4A	Regulation of KDM4A stability and target gene transcription	[47]
FBXL4	KDM4A	Regulation of KDM4A stability	[48]
β-TrCP1	UHRF1	Maintenance of DNA methylation patterns under DNA damage	[49]

## References

[1] Miller KD, Siegel RL, Lin CC, Mariotto AB, Kramer JL, Rowland JH, Stein KD, Alteri R, Jemal A (2016). Cancer treatment and survivorship statistics, 2016. CA Cancer J Clin.

[2] Shen J, Song G, An M, Li X, Wu N, Ruan K, Hu J, Hu R (2014). The use of hollow mesoporous silica nanospheres to encapsulate bortezomib and improve efficacy for non-small cell lung cancer therapy. Biomaterials.

[3] Song G, Shen J, Jiang F, Hu R, Li W, An L, Zou R, Chen Z, Qin Z, Hu J (2014). Hydrophilic molybdenum oxide nanomaterials with controlled morphology and strong plasmonic absorption for photothermal ablation of cancer cells. ACS Appl Mater Interfaces.

[4] Baylin SB, Jones PA (2011). A decade of exploring the cancer epigenome - biological and translational implications. Nat Rev Cancer.

[5] Shen J, Sheng X, Chang Z, Wu Q, Wang S, Xuan Z, Li D, Wu Y, Shang Y, Kong X, Yu L, Li L, Ruan K (2014). Iron metabolism regulates p53 signaling through direct heme-p53 interaction and modulation of p53 localization, stability, and function. Cell Rep.

[6] Dawson MA, Kouzarides T (2012). Cancer epigenetics: from mechanism to therapy. Cell.

[7] Rodriguez-Paredes M, Esteller M (2011). Cancer epigenetics reaches mainstream oncology. Nat Med.

[8] Tsai HC, Baylin SB (2011). Cancer epigenetics: linking basic biology to clinical medicine. Cell Res.

[9] Spruck CH, Strohmaier HM (2002). Seek and destroy: SCF ubiquitin ligases in mammalian cell cycle control. Cell Cycle.

[10] Klotz K, Cepeda D, Tan Y, Sun D, Sangfelt O, Spruck C (2009). SCF(Fbxw7/hCdc4) targets cyclin E2 for ubiquitin-dependent proteolysis. Exp Cell Res.

[11] Zheng H, Shen M, Zha YL, Li W, Wei Y, Blanco MA, Ren G, Zhou T, Storz P, Wang HY, Kang Y (2014). PKD1 phosphorylation-dependent degradation of SNAIL by SCF-FBXO11 regulates epithelial-mesenchymal transition and metastasis. Cancer Cell.

[12] Wu W, Ding H, Cao J, Zhang W (2015). FBXL5 inhibits metastasis of gastric cancer through suppressing Snail1. Cell Physiol Biochem.

[13] Zhao J, Mialki RK, Wei J, Coon TA, Zou C, Chen BB, Mallampalli RK, Zhao Y (2013). SCF E3 ligase F-box protein complex SCF(FBXL19) regulates cell migration by mediating Rac1 ubiquitination and degradation. FASEB J.

[14] Chen BB, Coon TA, Glasser JR, Zou C, Ellis B, Das T, McKelvey AC, Rajbhandari S, Lear T, Kamga C, Shiva S, Li C, Pilewski JM (2014). E3 ligase subunit Fbxo15 and PINK1 kinase regulate cardiolipin synthase 1 stability and mitochondrial function in pneumonia. Cell Rep.

[15] Chan CH, Li CF, Yang WL, Gao Y, Lee SW, Feng ZZ, Huang HY, Tsai KKC, Flores LG, Shao YP, Hazle JD, Yu DH, Wei WY (2012). The Skp2-SCF E3 Ligase Regulates Akt Ubiquitination, Glycolysis, Herceptin Sensitivity, and Tumorigenesis. Cell.

[16] Shaik S, Nucera C, Inuzuka H, Gao D, Garnaas M, Frechette G, Harris L, Wan L, Fukushima H, Husain A, Nose V, Fadda G, Sadow PM (2012). SCF(beta-TRCP) suppresses angiogenesis and thyroid cancer cell migration by promoting ubiquitination and destruction of VEGF receptor 2. J Exp Med.

[17] Cassavaugh JM, Hale SA, Wellman TL, Howe AK, Wong C, Lounsbury KM (2011). Negative regulation of HIF-1alpha by an FBW7-mediated degradation pathway during hypoxia. J Cell Biochem.

[18] Inuzuka H, Tseng A, Gao D, Zhai B, Zhang Q, Shaik S, Wan L, Ang XL, Mock C, Yin H, Stommel JM, Gygi S, Lahav G (2010). Phosphorylation by casein kinase I promotes the turnover of the Mdm2 oncoprotein via the SCF(beta-TRCP) ubiquitin ligase. Cancer Cell.

[19] Inuzuka H, Shaik S, Onoyama I, Gao D, Tseng A, Maser RS, Zhai B, Wan L, Gutierrez A, Lau AW, Xiao Y, Christie AL, Aster J (2011). SCF(FBW7) regulates cellular apoptosis by targeting MCL1 for ubiquitylation and destruction. Nature.

[20] Dehan E, Bassermann F, Guardavaccaro D, Vasiliver-Shamis G, Cohen M, Lowes KN, Dustin M, Huang DC, Taunton J, Pagano M (2009). betaTrCP- and Rsk1/2-mediated degradation of BimEL inhibits apoptosis. Mol Cell.

[21] Chen ZW, Liu B, Tang NW, Xu YH, Ye XY, Li ZM, Niu XM, Shen SP, Lu S, Xu L (2014). FBXL5-mediated degradation of single-stranded DNA-binding protein hSSB1 controls DNA damage response. Nucleic Acids Res.

[22] Zhang YW, Brognard J, Coughlin C, You Z, Dolled-Filhart M, Aslanian A, Manning G, Abraham RT, Hunter T (2009). The F box protein Fbx6 regulates Chk1 stability and cellular sensitivity to replication stress. Mol Cell.

[23] Lu Y, Li J, Cheng D, Parameswaran B, Zhang S, Jiang Z, Yew PR, Peng J, Ye Q, Hu Y (2012). The F-box protein FBXO44 mediates BRCA1 ubiquitination and degradation. J Biol Chem.

[24] Skowyra D, Craig KL, Tyers M, Elledge SJ, Harper JW (1997). F-box proteins are receptors that recruit phosphorylated substrates to the SCF ubiquitin-ligase complex. Cell.

[25] Wu X, Johansen JV, Helin K (2013). Fbxl10/Kdm2b recruits polycomb repressive complex 1 to CpG islands and regulates H2A ubiquitylation. Mol Cell.

[26] Janzer A, Stamm K, Becker A, Zimmer A, Buettner R, Kirfel J (2012). The H3K4me3 Histone Demethylase Fbxl10 Is a Regulator of Chemokine Expression, Cellular Morphology, and the Metabolome of Fibroblasts. Journal of Biological Chemistry.

[27] Andersson A, Ritz C, Lindgren D, Eden P, Lassen C, Heldrup J, Olofsson T, Rade J, Fontes M, Porwit-Macdonald A, Behrendtz M, Hoglund M, Johansson B (2007). Microarray-based classification of a consecutive series of 121 childhood acute leukemias: prediction of leukemic and genetic subtype as well as of minimal residual disease status. Leukemia.

[28] Korkola JE, Houldsworth J, Chadalavada RS, Olshen AB, Dobrzynski D, Reuter VE, Bosl GJ, Chaganti RS (2006). Down-regulation of stem cell genes, including those in a 200-kb gene cluster at 12p13.31, is associated with in vivo differentiation of human male germ cell tumors. Cancer Research.

[29] Tzatsos A, Paskaleva P, Ferrari F, Deshpande V, Stoykova S, Contino G, Wong KK, Lan F, Trojer P, Park PJ, Bardeesy N (2013). KDM2B promotes pancreatic cancer via Polycomb-dependent and -independent transcriptional programs. J Clin Invest.

[30] Kottakis F, Foltopoulou P, Sanidas I, Keller P, Wronski A, Dake BT, Ezell SA, Shen Z, Naber SP, Hinds PW, McNiel E, Kuperwasser C, Tsichlis PN (2014). NDY1/KDM2B Functions as a Master Regulator of Polycomb Complexes and Controls Self-Renewal of Breast Cancer Stem Cells. Cancer Research.

[31] Ueda T, Nagamachi A, Takubo K, Yamasaki N, Matsui H, Kanai A, Nakata Y, Ikeda K, Konuma T, Oda H, Wolff L, Honda Z, Wu X (2015). Fbxl10 overexpression in murine hematopoietic stem cells induces leukemia involving metabolic activation and upregulation of Nsg2. Blood.

[32] Chen S, Chen J, Zhan Q, Zhu Y, Chen H, Deng X, Hou Z, Shen B, Chen Y, Peng C (2014). H2AK119Ub1 and H3K27Me3 in molecular staging for survival prediction of patients with pancreatic ductal adenocarcinoma. Oncotarget.

[33] Blackledge NP, Farcas AM, Kondo T, King HW, McGouran JF, Hanssen LL, Ito S, Cooper S, Kondo K, Koseki Y, Ishikura T, Long HK, Sheahan TW (2014). Variant PRC1 complex-dependent H2A ubiquitylation drives PRC2 recruitment and polycomb domain formation. Cell.

[34] Tzatsos A, Pfau R, Kampranis SC, Tsichlis PN (2009). Ndy1/KDM2B immortalizes mouse embryonic fibroblasts by repressing the Ink4a/Arf locus. Proc Natl Acad Sci U S A.

[35] Tzatsos A, Paskaleva P, Lymperi S, Contino G, Stoykova S, Chen Z, Wong KK, Bardeesy N (2011). Lysine-specific demethylase 2B (KDM2B)-let-7-enhancer of zester homolog 2 (EZH2) pathway regulates cell cycle progression and senescence in primary cells. J Biol Chem.

[36] Shen J, Zhang S, Li Y, Zhang W, Chen J, Zhang M, Wang T, Jiang L, Zou X, Wong J, Li X, Cui Y, Wang C (2011). p14(ARF) inhibits the functions of adenovirus E1A oncoprotein. Biochem J.

[37] He J, Anh TN, Zhang Y (2011). KDM2b/JHDM1b, an H3K36me2-specific demethylase, is required for initiation and maintenance of acute myeloid leukemia. Blood.

[38] Blackledge NP, Zhou JC, Tolstorukov MY, Farcas AM, Park PJ, Klose RJ (2010). CpG islands recruit a histone H3 lysine 36 demethylase. Mol Cell.

[39] Wagner KW, Alam H, Dhar SS, Giri U, Li N, Wei Y, Giri D, Cascone T, Kim JH, Ye Y, Multani AS, Chan CH, Erez B (2013). KDM2A promotes lung tumorigenesis by epigenetically enhancing ERK1/2 signaling. J Clin Invest.

[40] Dhar SS, Alam H, Li N, Wagner KW, Chung J, Ahn YW, Lee MG (2014). Transcriptional repression of histone deacetylase 3 by the histone demethylase KDM2A is coupled to tumorigenicity of lung cancer cells. J Biol Chem.

[41] Kawakami E, Tokunaga A, Ozawa M, Sakamoto R, Yoshida N (2015). The histone demethylase Fbxl11/Kdm2a plays an essential role in embryonic development by repressing cell-cycle regulators. Mech Dev.

[42] Pedersen MT, Helin K (2010). Histone demethylases in development and disease. Trends Cell Biol.

[43] Cardamone MD, Tanasa B, Chan M, Cederquist CT, Andricovich J, Rosenfeld MG, Perissi V (2014). GPS2/KDM4A pioneering activity regulates promoter-specific recruitment of PPARgamma. Cell Rep.

[44] Bouzas SO, Marini MS, Torres Zelada E, Buzzi AL, Morales Vicente DA, Strobl-Mazzulla PH (2016). Epigenetic activation of Sox2 gene in the developing vertebrate neural plate. Mol Biol Cell.

[45] Wu L, Wary KK, Revskoy S, Gao X, Tsang K, Komarova YA, Rehman J, Malik AB (2015). Histone Demethylases KDM4A and KDM4C Regulate Differentiation of Embryonic Stem Cells to Endothelial Cells. Stem Cell Reports.

[46] Guerra-Calderas L, Gonzalez-Barrios R, Herrera LA, Cantu de Leon D, Soto-Reyes E (2015). The role of the histone demethylase KDM4A in cancer. Cancer Genet.

[47] Tan MK, Lim HJ, Harper JW (2011). SCF(FBXO22) regulates histone H3 lysine 9 and 36 methylation levels by targeting histone demethylase KDM4A for ubiquitin-mediated proteasomal degradation. Mol Cell Biol.

[48] Van Rechem C, Black JC, Abbas T, Allen A, Rinehart CA, Yuan GC, Dutta A, Whetstine JR (2011). The SKP1-Cul1-F-box and Leucine-rich Repeat Protein 4 (SCF-FbxL4) Ubiquitin Ligase Regulates Lysine Demethylase 4A (KDM4A)/Jumonji Domain-containing 2A (JMJD2A) Protein. Journal of Biological Chemistry.

[49] Chen H, Ma H, Inuzuka H, Diao J, Lan F, Shi YG, Wei W, Shi Y (2013). DNA damage regulates UHRF1 stability via the SCF(beta-TrCP) E3 ligase. Mol Cell Biol.

